# Supplementary material for: Sexual Victimization in LGB+ Persons in Belgium: Consequences, Help-Seeking Behavior, and Othering-Based Stress
Source: Healthcare (Basel). 2025 Oct 29;13(21):2744. doi: 10.3390/healthcare13212744 (PMC12608960; doi:10.3390/healthcare13212744)
Supplement: Supplementary file 1 [file healthcare-13-02744-s001.zip › healthcare-3877330-supplementary.pdf]

Supplementary material – SV LGB Belgium help-seeking De Schrijver et al

Supplementary material S1. Detailed outcome measurements sexual victimisation

"In the following part we will present a list of situations that may have happened to you."

- ⇨ Item is presented (cf. *infra*)
- ⇨ "Has this ever happened to you?"
    - o Yes
    - o No
- ⇨ If Yes: "How often has this happened to you in the past 12 months?"
    - o Daily
    - o Weekly
    - o Monthly
    - o Less than monthly
    - o Never

Presented items:

<u>Hands-off sexual victimisation (no physical contact):</u>

- *Sexual staring:* Someone stared at me in a sexual way or looked at my intimate body parts (e.g., breasts, vagina, penis, anus) when I didn't want it to happen.
- *Sexual innuendo:* Someone made teasing comments of a sexual nature about my body or appearance even though I didn't want it to happen.
- *Showing sexual images:* Someone showed me sexual or obscene materials such as pictures, videos, directly or over the internet (including email, social networks and chat platforms) even though I didn't want to look at them. This does not include mass mailings or spam.
- *Sexual calls or texts:* Someone made unwelcome sexual or obscene phone calls or texts to me.
- *Voyeurism:* I caught someone watching me, taking photos or filming me when I didn't want it to happen while I was undressing, nude or having sex.
- *Distribution of sexual images:* Someone distributed naked pictures or videos of me directly or over the internet (including email, social networks and chat platforms) when I didn't want it to happen.
- *Exhibitionism:* Someone showed their intimate body parts (e.g., breasts, vagina, penis, anus) to me in a sexual way and/or masturbated in front of me when I didn't want to see it.
- *Forcing to show intimate body parts:* Someone made me show my intimate body parts (e.g., breasts, vagina, penis, anus) online or face-to-face when I didn't want to do it.

<u>Hands-on sexual victimisation</u>

<u>Sexual abuse (physical contact but no penetration):</u>

- *Kissing:* Someone kissed me against my will.
- *Touching in care:* Someone touched my intimate body parts (e.g., breasts, vagina, penis, anus) during care against my will.
- *Fondling/rubbing:* Someone fondled or rubbed up against my intimate body parts (e.g., breasts, vagina, penis, anus) against my will.
- *Forced undressing:* Someone removed (some of) my clothes against my will.

<u>Rape and attempted rape (physical contact with attempted or completed penetration):</u>

- *Oral penetration:* Someone had oral sex with me or made me give oral sex against my will.

- *Attempt of oral penetration:* Someone tried, but did not succeed, to have oral sex with me or tried to make me give oral sex against my will.
- *Vaginal or anal penetration:* Someone put their penis, finger(s) or object(s) into my vagina or anus against my will.
- *Attempt of vaginal or anal penetration:* Someone tried, but did not succeed to put their penis, finger(s) or object(s) into my vagina or anus against my will.
- *Forcing to penetrate:* Someone made me put my penis, finger(s) or object(s) into their (or someone's) vagina or anus against my will.

Supplementary material S2. Othering-based stress scale (OBS-S)

Scoring: The othering-based stress scale is scored by averaging all the subscales. Subscales are scored by averaging all the items relevant to the subscale. The subscale 'Community connectedness' should be reversed before including into the total Othering-based stress score. Total OBS scores can range from 1 to 5, with higher scores indicating greater othering-based stress.

There are 2 versions of the OBS-S: one adapted for sexual and gender minorities; one for persons who belong to a minority group because of their ethnicity, skin colour, religion or philosophy of life

Introduction: *'You have indicated to have one or more characteristics that may distinguish you from the majority of the people in Belgium. This may make you part of a group of people with common interests or characteristics which distinguish them from the more numerous majority of the population of which they form a part. How frequently occur the situations described below in your life?'*

The OBS-S for sexual and gender minorities

Subscale	Item	Scoring
Identity concealment	• I avoid telling people about certain things in my life that might imply that I am LGBTQIA+<br>• I avoid talking about my love life because I do not want others to know that I am LGBTQIA+<br>• I do not bring a date to social events because I do not want others to know that I am LGBTQIA+	1= never<br>2= rarely<br>3= sometimes<br>4= regularly<br>5= all the time
Micro-aggressions	• I am expected to educate non-LGBTQIA+ people about LGBTQIA+ issues<br>• People have re-labelled my identity, or referred to me by a name / pronouns that are different from how I identify myself<br>• I have been accused of being too defensive or politically correct when talking about LGBTQIA+ issues with someone who is not LGBTQIA+	1= never<br>2= rarely<br>3= sometimes<br>4= regularly<br>5= all the time
Rejection anticipation	• When I meet someone new, I worry that they secretly don't like me because I am LGBTQIA+<br>• I brace myself to be treated disrespectfully because I am LGBTQIA+<br>• I expect that others will not accept me because I am LGBTQIA+	1= never<br>2= rarely<br>3= sometimes<br>4= regularly<br>5= all the time

Victimisation events	• I have been verbally harassed or called names because I am LGBTQIA+<br>• Others have threatened to harm me because I am LGBTQIA+<br>• I have been bullied by others because I am LGBTQIA+<br>• I have overheard people making negative remarks about LGBTQIA+ people<br>• I felt unsafe in a group of people because I am LGBTQIA+<br>• I have felt discriminated against on the labour and/or housing market because I am LGBTQIA+<br>• I have been physically attacked because I am LGBTQIA+<br>• I have had my personal property purposefully damaged by others because I am LGBTQIA+<br>• I have received unwanted sexual attention or been asked inappropriate questions about my sexual life because I am LGBTQIA+<br>• I have endured unwanted sexual contact because I am LGBTQIA+	1= never<br>2= rarely<br>3= sometimes<br>4= regularly<br>5= all the time
	To what extent do you agree with the following statements?	
Internalized stigma	• If I was offered the chance to be someone who is not LGBTQIA+, I would accept the opportunity<br>• I wish I wasn't LGBTQIA+<br>• I envy people who are not LGBTQIA+	1=strongly disagree<br>2=disagree<br>3=neither disagree nor agree<br>4= agree<br>5= strongly agree
Community connectedness	• I feel that I could find information and pamphlets on LGBTQIA+ issues<br>• I feel that I could find professional services for LGBTQIA+ issues if I needed to<br>• I feel that I could find a public space that is supportive of LGBTQIA+ activities	1=strongly disagree<br>2=disagree<br>3=neither disagree nor agree<br>4= agree<br>5= strongly agree

The OBS-S for persons who belong to a minority group because of their ethnicity, skin colour, religion or philosophy of life

Subscale	Item	Scoring
Identity concealment	• I avoid telling people about certain things in my life that might imply that I have a different skin colour, cultural or religious background<br>• I avoid talking about certain traditions in my life because I do not want others to know that I have a different skin colour, cultural or religious background<br>• I do not wear traditional clothing or symbols to social events because I do not want others to know that I have a different skin colour, cultural or religious background	1= never<br>2= rarely<br>3= sometimes<br>4= regularly<br>5= all the time
Micro-aggressions	• I am expected to explain or educate others about issues related to my skin colour, cultural or religious background<br>• People have re-labelled my identity, or referred to me by a name / pronouns that are different from how I identify myself<br>• I have been accused of being too defensive or politically correct when talking about issues related to my skin colour, cultural or religious background with someone who is not familiar with it	1= never<br>2= rarely<br>3= sometimes<br>4= regularly<br>5= all the time
Rejection anticipation	• When I meet someone new, I worry that they secretly don't like me because of my skin colour, cultural or religious background<br>• I brace myself to be treated disrespectfully because of my skin colour, cultural or religious background<br>• I expect that others will not accept me because of my skin colour, cultural or religious background	1= never<br>2= rarely<br>3= sometimes<br>4= regularly<br>5= all the time
Victimisation events	• I have been verbally harassed or called names because of my skin colour, cultural or religious background<br>• Others have threatened to harm me because of my skin colour, cultural or religious background<br>• I have been bullied by others because of my skin colour, cultural or religious background<br>• I have overheard people making negative remarks about people with another skin colour, cultural or religious background<br>• I felt unsafe in a group of people because of my skin colour, cultural or religious background<br>• I have felt discriminated against on the labour and/or housing market because of my skin colour, cultural or religious background<br>• I have been physically attacked because of my skin colour, cultural or religious background<br>• I have had my personal property purposefully damaged by others because of my skin colour, cultural or religious background<br>• I have received unwanted sexual attention or been asked inappropriate questions about my sexual life because of my skin colour, cultural or religious background<br>• I have endured unwanted sexual contact because of my skin colour, cultural or religious background	1= never<br>2= rarely<br>3= sometimes<br>4= regularly<br>5= all the time
	To what extent do you agree with the following statements?	
Internalized stigma	• If I was offered the chance to be someone with another skin colour, cultural or religious background, I would accept the opportunity<br>• I wish I had a different skin colour, cultural or religious background<br>• I envy people who don't have this skin colour, cultural or religious background	1=strongly disagree<br>2=disagree<br>3=neither disagree nor agree<br>4= agree

		5= strongly agree
Community connectedness	• I feel that I could find information and pamphlets on issues related to my skin colour, cultural or religious background<br>• I feel that I could find professional services for issues related to my skin colour, cultural or religious background, if I needed to<br>• I feel that I could find a public space that is supportive of activities for people with the same skin colour, cultural or religious background as me	1=strongly disagree<br>2=disagree<br>3=neither disagree nor agree<br>4= agree<br>5= strongly agree

Supplementary material S3. Table of sociodemographic characteristics of the quantitative sample

Table S1. Sample Composition (*n* = 4632). Socio-demographic Information and Number of Sexual Partners Presented for Heterosexual Participants and for Participants Who Self-identified as LGB+ (LGB+), as well as for LGB+ Who Reported Belonging to a Sexual Minority Group (Sexual Minority) and LGB+ That Did Not (Non-sexual Minority).

Variable	Within total sample (*n* = 4,632)		
	Heterosexual (*n* =4,168; 89.98%) n (Valid %)	LGB+ (*n* = 464; 10.02%) n (Valid %)	χ^2; df; p-value; V
**Sex assigned at birth**			2.27; 1; .132; .022
Female	2,083 (49.98)	249 (53.66)	
Male	2,085 (50.02)	215 (46.34)	
**Age groups** *[mean(SD)]*	*39.68 (17.12)*	*33.63 (15.11)*	8.07; 603; <.001; .357*
16-24 years old	1,254 (30.09)[a]	198 (42.67)[b]	
25-49 years old	1,374 (32.96)[a]	174 (37.50)[b]	
50-69 years old	1,540 (36.95)[a]	92 (19.83)[b]	
Educational level			10.44; 2; .005; .047
Primary education or none	255 (6.12)[a]	26 (5.60)[a]	
Secondary education	1,803 (43.26)[a]	237 (51.08)[b]	
Higher education	2,110 (50.62)[a]	201 (43.32)[b]	
Occupational status			25.39; 2; <.001; .074
Remunerated workforce	2,151 (51.61)[a]	196 (42.24)[b]	
Student	1,034 (24.81)[a]	164 (35.34)[b]	
Other	983 (23.58)[a]	104 (22.41)[a]	
Financial situation			20.32; 1; <.001; .066
Perceived as difficult	3,101 (74.40)	300 (64.66)	
Perceived as easy	1,067 (25.60)	164 (35.34)	
Gender			<.001°
Man[1]	2,076 (49.81)	206 (44.40)	
Woman[1]	2,083 (49.98)	233 (50.21)	
Trans man	0	5 (1.08)	
Trans woman	0	1 (0.22)	
Other	9 (0.22)	19 (4.09)	
Number of sexual partners			159.92; 5; <.001; .186
0	565 (13.56)[a]	97 (20.90)[b]	
1 to 2	1406 (33.73)[a]	81 (17.46)[b]	
3 to 7	1274 (30.57)[a]	115 (24.78)[b]	
8 to 20	688 (16.51)[a]	90 (19.40)[a]	
21 to 100	210 (5.04)[a]	62 (13.36)[b]	
> 100	25 (0.60)[a]	19 (4.09)[b]	

Note: Because the comparisons in this table involved 2 sets of 7 independent tests, we adopted a Bonferroni-corrected significance level of .05/7 = .007 for these two sets of analyses. *Abbreviations*: LGB+ = lesbian, gay, bisexual, pan-

/omnisexual, asexual, other; V = Cramer's V (cutoffs 0.10, 0.30, and 0.50 are recommended to be considered small, medium, and large in magnitude, respectively).

* Independent sample t-test with equal variances not assumed (instead of chi-square-test): t; df; p-value; Cohen's d (cutoffs 0.10, 0.30, and 0.50 are recommended to be considered small, medium, and large in magnitude, respectively).

° Fisher's Exact Test (instead of Chi Square Test): p-value

[1] For inclusivity reasons, non-cisgender identifying persons could also indicate 'man' and 'woman' as gender identity.

[a,b] Each subscript letter denotes a subset of the variables' categories whose column proportions do not differ significantly from each other (post-hoc $\chi 2$ test $p > 0.05$) between Heterosexual and LGB+ participants. If both cells on the same row have subscript "a" it means they do not differ significantly; if the first cell has subscript "a" and the second cell subscript "b" they do differ significantly for the corresponding category of the variable of that specific row.

Source: De Schrijver, L.; Fomenko, E.; Motmans, J.; Janssen, E.; Krahé, B.; Roelens, K.; Vander Beken, T.; Keygnaert, I. Sexual violence in lgb+ persons in Belgium: results from the UN-MENAMAIS study in Belgium. Proceedings of the International Society for Research on Agression (ISRA) XXIV World Meeting, Canada, 22 July **2022**
